# Understanding the Landscape of Consultation Liaison Psychologists in Academic Medical Centers

**DOI:** 10.1007/s10880-024-10018-4

**Published:** 2024-05-03

**Authors:** Caitlin A. LaGrotte, Anastasia Bullock, Corey Doremus, Carissa Aricola

**Affiliations:** Department of Medicine, Cooper University Health Care and Cooper Medical School of Rowan University, Camden, NJ USA

**Keywords:** Psychology, Consultation-liaison, Clinical practice, Organizational structure

## Abstract

Current literature lacks data related to the role of psychologists on consultation-liaison (CL) services; previous data indicates only 4% of CL services are run by psychologists, while 32% of liaison mental health services include a psychologist. As CL psychologists’ roles within hospitals grow, it is critical to identify clinical strategies and organizational structures of CL services across hospital systems. The current study seeks to provide a deeper understanding of CL psychologists’ scope of work. Participants (*N* = 77) (15% response rate) completed a measure developed for this study, exploring psychologist roles, clinical practice, and departmental structures. Thirty-two percent of respondents were in Psychiatry Departments, 58% were in academic medical centers, almost half had training programs and the most frequently utilized billing code was**:** Given the limited data available, this study provided a contemporary and foundational understanding of the CL psychologist roles as well as future avenues of empirical inquiry such as discrete organization and structural characteristics.

## Introduction

Within a medical setting, psychological or psychiatric consultation-liaison (CL) teams are consulted for a wide range of diagnostic and management reasons, including assessment, interventions for mood disorders/anxiety, pain, coping/adjustment, and behavioral/conduct concerns (Carter et al., 2003; Kitts et al., 2013). As a field, the role of psychologists in academic health centers is growing; however, there is a lack of data related to psychologists’ role on CL services and an inadequate understanding of how CL services operate and provide care (e.g., assessment and treatment) to their patients. Previous research suggests that only 4% of CL services are independently run by psychologists while just 32% of liaison mental health service teams include a psychologist (Callaghan et al., 2003). Extant research has focused on individual sites publishing on their internal program development (Lechner & Stucky, 2000), which has provided a general roadmap for those creating programs. However, most literature in this area is almost two decades old and has primarily been focused on psychiatric CL services which somewhat limits its utility to psychologists.

More recent publications have attempted to fill this void by focusing on satisfaction and success of psychological CL services (e.g., Bullock et al., 2021). Specifically, it has been found that interdisciplinary teams who utilized psychological services believed there was an improvement of patient care, efficiency, and provider satisfaction because of this service (Agapoff et al., 2020). Further, medical teams within the hospital are often met with patients suffering from an array of mental health disorders that fundamentally interfere with their effective participation in care. CL psychologists provide specialized assessment and intervention to provide accurate diagnosis and therapeutic cognitive and behavioral interventions, which supports both the medical teams and patients (Scott et al., 2021). An additional benefit of hospital-based psychological CL services is their ability to provide efficacious violence prevention and verbal de-escalation training to medical staff to support health systems’ ability to address behavioral disruptions. Yost et al. (2021) found that directly training staff in these skills led to a decrease in the number of acute behavioral interventions needed by as much as 50%, as well as increased confidence of staff working with behaviorally complex patients. Despite limitations and the growing number of consultations during the COVID-19 pandemic (e.g., virtual service delivery; Leith et al., 2022), patients reported satisfaction with psychological CL services (Steinberg et al., 2021). However, despite more recent efforts to add to the limited existing literature there continues to be a lack of global understanding of CL psychologists’ approach and scope of work.

This exploratory study seeks to provide a more global understanding of CL psychologists’ scope of work and clinical practice. It is timely to explore these factors and update the literature in this area due to the continued and rapid expansion of psychologists' presence in academic medical centers. Establishing a better understanding of the field of CL psychology will allow for further growth as well as the creation of standards of care or recommended practices across settings.

## Method

At the time of recruitment there was no centralized list of psychologists employed on consultation-liaison services, so investigators attempted to find them through three recruitment strategies. The study advertisement was distributed via the APAHC (Association of Psychologists in Academic Health Centers) member listserv (*n* = 418), to training directors of psychology internship programs with hospital-based rotations (*n* = 384), and to those advertising a consultation-liaison position on professional listservs (*n* = 27).

Inclusion criteria included being a licensed clinical psychologist on a CL service. Recruitment occurred from August 16, 2021, to April 13, 2022, as after this date no additional responses were received for approximately one month and there were no further recruitment avenues. Approximately 9% of the individuals contacted completed the study. Participants provided consent and completed a one-time, online survey via Research Electronic Data Capture (REDCap), a secure online database (Harris et al., 2009, 2019). Participants were assigned and identified by an arbitrary research ID number that was used throughout the duration of the study.

The present study utilized a survey constructed to assess the organizational structure of the participant’s CL service, relevant roles and responsibilities, areas of clinical practice, consult characteristics, and aspects of job satisfaction. For the development of the survey, psychologists who had been in the field of CL psychology for numerous years and had practiced in different CL models of care were approached for assistance in questionnaire review. These individuals were identified through longstanding involvement both in professional organizations and training programs. Reviewers were sent the questionnaire along with the rationale for the study and they provided recommended revisions to the study questionnaire. Several potentially salient dimensions of CL programs were identified at this point, including service utilization across hospital departments, structural characteristics of the service itself, and numerous other domains. The study was approved by the Institutional Review Board at [Name withheld for peer review].

## Results

Of the 77 participants, 39 (72%) identified as women, and participant ages ranged from 25–74 years old. 85% of participants fell within the 25–54 year range. This sample largely identified as White (58%), and 31% of participants declined to indicate their race or ethnicity. Individuals who responded are employed either part-time (34%) or full-time (39%) on CL services at academic medical centers in the United States. Additional demographic information for the study can be found in Table 1.

**Table 1:** Demographic Information

Variable	Response	N(%)
Age	25–34	16 (29%)
	35–44	18 (33%)
	45–54	13 (24%)
	55–64	5 (9%)
	65–74	4 (7%)
	75 +	0 (0%)
	Declined to respond	21 (27%)
Gender	Genderqueer	0 (0%)
	Gender Variant, Gender Non-conforming	0 (0%)
	Man	15 (19%)
	Transgender Man/Trans Man	0 (0%)
	Transgender Woman/Trans Woman	0 (0%)
	Woman	39 (51%)
	Declined to respond	23 (30%)
Race/Ethnicity	Black	0 (0%)
	Asian	4 (5%)
	Latinx	3 (4%)
	Middle Eastern	0 (0%)
	Native American	1 (1%)
	Native Hawaiian	0 (0%)
	White	45 (58%)
	Declined to respond	24 (31%)
Marital Status	Single	12 (22%)
	Married	33 (60%)
	Partnered	7 (13%)
	Divorced	3 (5%)
	Separated	1 (2%)
	Widowed	0 (0%)
	Declined to respond	21 (27%)
Have Children	No	33 (43%)
	Yes	21 (27%)
	Declined to respond	2 (3%)
Full-Time Psychologists	0	17 (22%)
	1	17 (22%)
	2	15 (20%)
	3	3 (4%)
	4	2 (3%)
	5 +	4 (5%)
Part-Time Psychologists	0	23 (30%)
	1	12 (16%)
	2	8 (10%)
	3	7 (9%)
	4	3 (4%)
	5	1 (1%)
	6	1 (1%)
	8	1 (1%)
	10 +	2 (3%)
Training Orientation	Cognitive-Behavioral	39 (51%)
	Multiple	12 (16%)
	Psychodynamic/Psychoanalysis	3 (4%)
	Eclecticism	2 (3%)
	Humanistic and Person-Centered	1 (1%)
	Acceptance and Commitment	1 (1%)
Practice Orientation	Cognitive-Behavioral	54 (70%)
	Acceptance and Commitment	36 (47%)
	Motivational Interviewing	35 (46%)
	Dialectical-Behavioral	15 (20%)
	Behaviorism	14 (18%)
	Humanistic and Person-Centered	11 (14%)
	Psychodynamic/Psychoanalysis	5 (7%)

The most frequently reported departments that CL psychologists organizationally work within were Psychiatry (32%), Behavioral Health (or similar; 8%), and Hospital Medicine (5%). For the number of full- and part-time psychologists on a CL service, the average number of full-time psychologists was 1–2, while part-time was generally 2–3. Respondents worked in various settings including VA medical centers (7%), academic medical centers (58%), and community hospitals (10%). These facilities were based in rural (10%), suburban (14%), or urban (51%) locales. Of respondents, 47% percent described their CL service to be independently run by psychologists. Those who described their CL service as interdisciplinary reported the following specialties on their team: Psychiatrists (26%), Advanced Practice Providers (APNs or PAs; 13%), Social Workers (9%), and RNs (7%). Almost half of the respondents (49%) indicated they had internship and postdoctoral training programs with the most reported orientation being cognitive-behavioral (51%). Additional characteristics of CL services are represented in Tables 2 and 3. Hospital Medicine (42%), Adult Critical Care (34%), and Adult Trauma (30%) were the departments identified to consult CL psychologists the most.

**Table 2:** CL Service Characteristics

Variable	Response	N (%)
Full-Time Psychologists	1	17 (22%)
	2	15 (20%)
	3	3 (4%)
	4	2 (3%)
	5 +	4 (5%)
Part-Time Psychologists	0	23 (30%)
	1	12 (16%)
	2	8 (10%)
	3	7 (9%)
	4	3 (4%)
	5 +	5 (7%)
	Both	20 (26%)
Hospital Location	Rural	8 (10%)
	Suburban	11 (14%)
	Urban	39 (51%)
Hospital Type	Academic	45 (58%)
	Community	8 (10%)
	Other	5 (7%)
Hospital Beds	< 100	5 (7%)
	100–499	24 (31%)
	500–999	21 (27%)
	1000–1999	5 (7%)
	2000 +	3 (4%)
Departments with dedicated CL Psychologist	Hospital Medicine/Internal Medicine	16 (21%)
	Adult Critical Care/Intensive Care	15 (19%)
	Oncology	14 (18%)
	Additional Response	14 (18%)
	Trauma Surgery	13 (17%)
	Transplant	13 (17%)
	Psychiatry	11 (14%)
	Bariatric and Metabolic Surgery	10 (13%)
	Emergency Medicine	10 (13%)
	Family Medicine	10 (13%)
	Pediatrics	9 (12%)
	Cardiology	8 (10%)
	Addiction Medicine	6 (8%)
	Ob/Gyn Women’s Health	6 (8%)
	Adolescent Medicine	5 (6%)
	Neurology	5 (6%)
	Anesthesiology	4 (5%)
	General Surgery	4 (5%)
	Endocrinology	3 (4%)
	Pediatric Critical Care	2 (3%)
	Orthopedic Surgery	2 (3%)
	Urology	1 (1%)
Home Department	Psychiatry	25 (33%)
	Additional Response:	21 (27%)
	Hospital Medicine / Internal Medicine	4 (5%)
	Trauma Surgery	3 (4%)
	Pediatrics	2 (3%)
	Critical Care / Intensive Care-Adult	1 (1%)
	Family Medicine	1 (1%)
	Oncology	1 (1%)
Trainees Present	Externs	25 (33%)
	Interns	38 (49%)
	Postdocs	38 (49%)

**Table 3:** Consult Reasons, Billing, and Assessments

Variable	Response	N
Consult Reasons	Adjustment to Illness	40 (52%)
	Depression	38 (49%)
	Anxiety	35 (45%)
	Acute Stress/Trauma	30 (39%)
	Coping	26 (34%)
	Capacity	16 (21%)
	Suicidality/Crisis/Risk Management Concerns	16 (21%)
	Disruptive Behaviors (physical, inappropriate behaviors)	14 (18%)
	Alcohol Use	12 (16%)
	Neurocognitive deficits	11 (14%)
	Chronic pain	10 (13%)
	Pain management (acute	10 (13%)
	Medication or treatment non-compliance	9 (12%)
	Substance Use	8 (10%)
	End of life	7 (9%)
	Family Support	6 (8%)
	New diagnosis	6 (8%)
	Grief	5 (6%)
	Additional Response:	4 (5%)
	Amputation	3 (4%)
	Disordered Eating	3 (4%)
	Domestic violence	3 (4%)
	Insomnia	3 (4%)
	Sleep disturbance	3 (4%)
	Somatic Symptom Disorder	3 (4%)
	Non-suicidal self-injurious behavior	2 (3%)
	Conversion Disorder	1 (1%)
	Malingering	1 (1%)
	Post-partum	1 (1%)
	Specific phobia (e.g., needles)	1 (1%)
Billing Codes	Psychodiagnostic exam	38 (49%)
	Individual therapy 30 min	34 (44%)
	Individual therapy 45 min	25 (32%)
	Health and Behavior Assessment / Re-assessment	20 (26%)
	Individual therapy 60 min	17 (22%)
	Health and Behavior Individual face to face	14 (18%)
	Family therapy with patient present	9 (12%)
	Health and Behavior Family with patient present	8 (10%)
	Health and Behavior Family no patient present	5 (6%)
	Psychotherapy for CRISIS	5 (6%)
	Family therapy without patient present	4 (5%)
	Group therapy	2 (3%)
Assessments	Patient Health Questionnaire	37 (48%)
	Generalized Anxiety Disorder 7-item	33 (43%)
	Montreal Cognitive Assessment	33 (43%)
	Alcohol Use Disorders Identification Test	19 (25%)
	Posttraumatic Stress Disorder Checklist	17 (22%)
	Patient Health Questionnaire-2	16 (21%)
	Beck Depression Inventory	13 (17%)
	Beck Anxiety Inventory	11 (14%)
	Hospital Anxiety and Depression Scale	10 (13%)
	Center for Epidemiological Studies Depression Scale	5 (6%)
	Edinburgh Postnatal Depression Scale	3 (4%)
	Impact of Events Scale	2 (3%)
	Beck Scale for Suicidal Ideation	1 (1%)
	Cannabis Use Disorders Identification Test	1 (1%)
	National Institute on Drug Abuse Quick Screen	1 (1%)

CL services were noted to address ongoing treatment referrals in several different ways. Outpatient psychotherapy referrals were identified to be handled through internal sources, either within the same department (62%) or through a different department (33%). However, external referrals to community treatment also frequently occurred (62%). Inpatient psychiatric referrals were either internal (44%) or external (43%). For billing codes, psychodiagnostic exam (49%), individual therapy 30 (44%) and 45 (32%) minutes, and health and behavior assessment (26%) were reported to be used most often. The most common inappropriate referrals included risk-related concerns that were more appropriate for Psychiatry (21%), medication management (12%), housing instability (9%), and unrecognized delirium (6%).

## Discussion

The present study sought to better understand and delineate the characteristics of the practice of CL psychology as there is no real current sense of how CL psychology is distributed through medical systems. Over the past twenty years there has been an increase in CL psychology, both in the number of positions as well as the number of CL services run independently by psychologists (47% vs 4%; Callaghan et al., 2003). Results also suggest that specific medical departments are appreciating the need for a CL psychologist dedicated to their patient population and/or unit. Departments most frequently engaging with CL consults (Hospital Medicine, Adult Critical Care, and Adult Trauma) were likewise identified as some of the departments who have hired a dedicated CL psychologist to exclusively see their patients. Robust data tracking and management is common practice amongst CL psychologists, often focusing on the number and distribution of consults over a specific period of time. Relevant consult characteristics include consulting department, referral reason, and ultimate billing codes. Presenting this information to hospital administration can support the rationale for creating additional positions, as well as identify key stakeholders (e.g., physician champions) who can speak to how these clinical needs are met by psychologists.

A majority of the sample (51%) reported their training orientation as cognitive-behavioral, which has been the predominant orientation over the past two decades. This is unsurprising given that 85% of the sample was between the ages of 25–54. Years of empirical literature support the use of cognitive-behavioral interventions for patient’s experience of comorbid psychological and medical conditions, which is reflected in how respondents practice (Andersen, 2003; Ehlert et al., 1999; Gorsky et al., 2021; Lackner et al., 2004; Vanhaudenhuyse et al., 2017). A growing body of literature suggests utilizing Acceptance and Commitment Therapy (ACT) interventions for this population (Feliu-Soler et al., 2018; Rashidi et al., 2021). This approach is intuitive as there are many situations in a medical hospital that are outside of a patient’s control leading to interventions to refocus attention to what is meaningful or valued in their lives. Commonly reported assessments, which include the PHQ-9 (Patient Health Questionnaire – 9; Kroenke et al., 2001), GAD-7 (Generalized Anxiety Disorder – 7; Spitzer et al., 2006), and MoCA (Montreal Cognitive Assessment; Nasreddine et. al, 2005), are largely concerned with either specific clinical syndromes or discrete symptoms and are categorically brief in delivery. This is likely a product of the focused and dynamic nature of CL services. Two of the three most common reasons for CL service consults in the present study were for anxiety and depression, which appears to be consistent with trends among US prevalence rates (Kessler et al., 2005; Lee et al., 2023). A potential factor in the prevalence of these consults is the high visibility of their symptoms, specifically in the context of a patient’s engagement with their care. Notably, a large percentage of respondents had training programs at their institutions. Training programs that include clinical experiences on CL services can enable further growth of CL psychologists in AHCs with the competencies required for CL psychology practice. Based on the responses, an area of growth may be expanding CL services to hospitals that serve rural communities, as only a small percentage of respondents were working in those communities. Growth in AHCs can be difficult because health care administrations measure cost effectiveness by requiring data on productivity (e.g., relative value units (RVUs), patient access (e.g., patient show rates, waiting lists), and other measurements of value added with respect to the cost of hiring. Differences across jurisdictions (e.g., state and organizational levels) also impact generalizability of how each provider, department, and organization can bill for services. Rather than reliance on mirroring exact policies and practices across CL psychology teams, it may be more beneficial to focus efforts toward sharing important components of the processes CL psychologists take to advocate for growth within their respective institutions (e.g., identifying key stakeholders, trialing services at no cost with support from the training program, working with billing and compliance to ensure understanding of documentation). The use of national organizations can be useful for collaboration and cross-system education about examples of successful processes, advocacy, and expansion efforts. Relevant organizations include the American Psychological Association, respective divisions (e.g., Division 38, Society for Health Psychology; Division 12, Society for Clinical Psychology), and special interest groups or sections (e.g., consultation-liaison interest group; Section 8, APAHC).

When considering the study’s results, a few factors should be taken into consideration. First, due to the sample size and numerous respondents who chose not to provide their demographic information, it cannot be determined how representative this sample is of the entire population of CL psychologists. Furthermore, there is limited literature to strengthen or contradict the results provided by respondents. Additionally, the survey response rate was 9%. While a limitation and relatively low, this response rate was consistent with response rates from 2008 and 2015 data that was published by the APA Center for Workforce Studies (Leventhal et al., 2021; Michalski & Kohout, 2011). Leventhal et al. (2021) note that response rates have been declining for some time and may not indicate a biased sample. At the time of recruitment, there was not a centralized list or formal professional group of CL psychologists, so the best possible efforts were made to maximize outreach to potential participants. It is important to acknowledge that certain aspects of the present recruitment mechanism (i.e., utilizing a list of psychology internship programs with hospital-based rotations) may have resulted in sampling bias. Since data collection has ended, a special interest group has been formed within APA Division 38 for CL psychologists, which should be utilized in future recruitment efforts. The study utilized self-report demographic data, which may contribute to recency biases when responding to items as well as potential inaccuracy when relying on participant memory and perspective of number and types of consults. It would be methodologically difficult to obtain objective data from medical records and billing departments across the country. Further, as any given medical system is likely to have differing internal structural characteristics (e.g., consult mechanisms, electronic medical record systems, methods of interdisciplinary communication) the self-reporting of personal experiences most directly accesses these factors. Lastly, by providing anonymity, multiple respondents from one institution may have responded to the survey; however, of those responding few had multiple psychologists on their service which might limit the error introduced.

The goal of the present study was to provide a general understanding of the roles and practices of psychologists on CL services, as well as where they fit departmentally across institutions. Future research should consider alternative data acquisition strategies across institutions to both increase objective accuracy and provide more granular details (e.g., frequency of consulting departments). The broad use of electronic medical records would ostensibly make such a strategy possible, if not simple. Specifically, multi-site studies that include different organizational models of CL psychologists would add a deeper understanding of these questions. Future CL psychology research could expand upon these findings to implement and measure targeted time-limited interventions to address the most commonly presenting concerns in an inpatient medical context. This paper strives to provide updated data on the current landscape of CL psychology. This was done through an examination of psychologists’ scope, organization, and structure within institutions as well as specific clinical practices to support the expansion and growth of these services.

## Data Availability

Data can be provided by the corresponding author upon request.
